# Supplementary material for: Cholinergic deficits selectively boost cortical intratelencephalic control of striatum in male Huntington’s disease model mice
Source: Nat Commun. 2023 Mar 14;14:1398. doi: 10.1038/s41467-023-36556-3 (PMC10011605; doi:10.1038/s41467-023-36556-3)
Supplement: Supplementary file 1 — Supplementary Information [file 41467_2023_36556_MOESM1_ESM.pdf]

Supplementary figures

Figure S1 - Pancani et al.

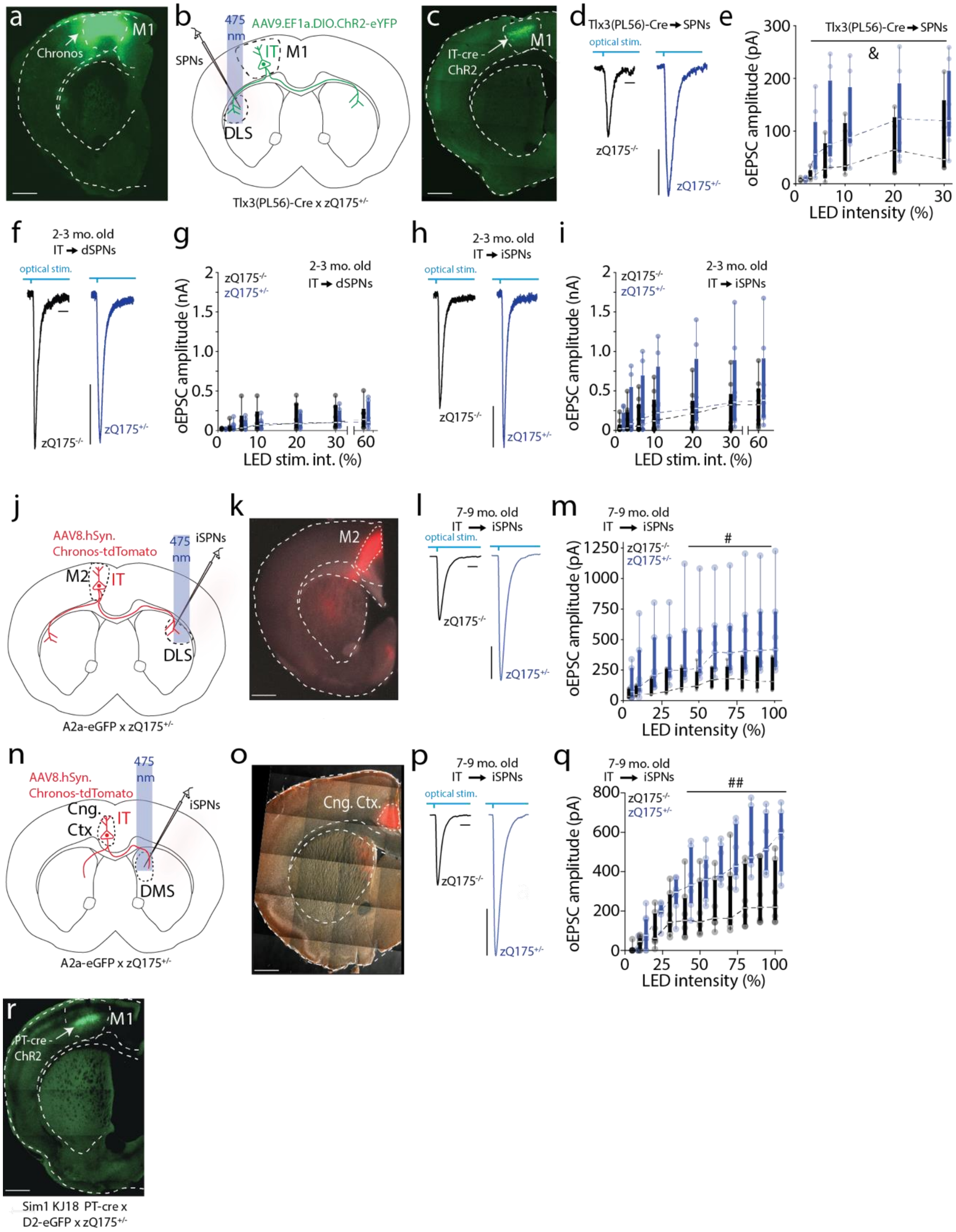

Figure S1 (a) Confocal image showing AAV9.hSyn.Chronos-GFP expression in primary motor cortex (M1; N=12). **(b)** Schematics of AAV9.EF1a.DIO.ChR2-eYFP injected in M1 in Tlx3(PL56)-Cre x zQ175$^{+/-}$ mice. **(c)** Confocal image showing AAV9.EF1a.DIO.ChR2-eYFP expression in intratelencephalic (IT) neurons (N=5). **(d)** Representative traces of optically evoked (oEPSCs) obtained by stimulating intratelencephalic (IT) axons and recorded in spiny projection neurons (SPNs) in ipsilateral dorsolateral striatum (DLS) in 8-9 month old Tlx3(PL56)-Cre x zQ175$^{+/-}$ mice and zQ175$^{-/-}$ controls. **(e)** Input/output curves (I/O) in SPNs, zQ175$^{-/-}$ (N=2 mice; n=5 neurons) vs. zQ175$^{+/-}$ (N=3; n=7); Significant increase in oEPSCs amplitude in zQ175 (**&**, p=0.031 in a Wilcoxon Signed Rank Test between both groups). **(f, h)** Traces of oEPSC obtained by

stimulating IT axons recorded in direct/indirect pathway SPNs (dSPNs/iSPNs, in contralateral DLS; 2-3 month-old zQ175$^{+/-}$ mice vs. zQ175$^{-/-}$ controls. **(g, i)** I/O graphs showing similar oEPSCs amplitude in zQ175$^{+/-}$ mice compared zQ175$^{-/-}$ controls is dSPNs (**g**, zQ175$^{-/-}$ N=2 mice, n=6 neurons; zQ175$^{+/-}$ N=2, n=7) and iSPNs (zQ175$^{-/-}$ N=3 mice, n=9 neurons; zQ175$^{+/-}$, N=3, n=8). (**j-q**) Depiction of AAV8.hSyn.Chronos-tdTomato stereotaxic injection in secondary motor cortex (**j**, M2) and cingulate cortex (**n**, Cng.Ctx). **(k, o)** Confocal images showing M2 (**k**, N=14) and Cng.Ctx (**o**, N=14) expression of AAV8.hSyn.Chronos-tdTomato. **(l, p)** oEPSCs traces evoked in iSPNs in contralateral DLS by stimulating M2 (**l**) or Cng.Ctx (**p**) terminals in dorsomedial striatum (DMS). **(m, q)** Input/output curves (I/O) showing increase in oEPSCs amplitude measured in iSPNs from zQ175$^{+/-}$ compared to zQ175$^{-/-}$ mice from (**m**) M2 axons stimulation (zQ175$^{-/-}$ (N=7 mice; n=7 neurons) vs. zQ175$^{+/-}$ (N=7; n=7); p-value (LED Intensity, % of max): n.s. (5, 10, 20, 30, 40%), #, significant increase: 0.026(50%), 0.026(60%), 0.017(70%), 0.017(80%), 0.0175(90%), 0.0175(100%); in a Mann-Whitney non-parametric two-sided test). **(q)** Cng. Ctx axon stimulation (zQ175$^{-/-}$ (N=7mice; n=7neurons) vs. zQ175$^{+/-}$ (N=7; n=7); p-value (LED Intensity, % of max): n.s. (5, 10, 20, 30), ##, significant increase: 0.041(40%), 0.038(50%), 0.017(60%), 0.038(70%), 0.011(80%), 0.007(90%), 0.007(100%); in a Mann-Whitney non-parametric two-sided test). **(r)** Coronal hemi-slice expressing AAV9.EF1a.DIO.ChR2-GFP in cortical pyramidal tract (PT) neurons from Sim1-KJ18 PT-cre mice (N=13). Scale bars in all confocal images are 1mm. All mice were male. Box-plots represent median and interquartile range, whiskers min/max value.

Figure S2 - Pancani et al.

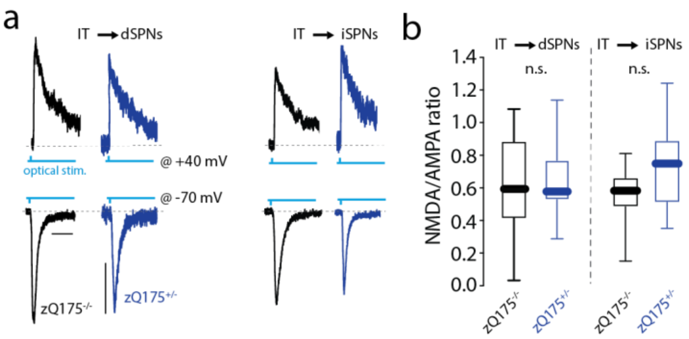

Figure S2 (a) Sample traces depicting synaptic NMDA and AMPA currents optically evoked stimulating intratelencephalic (IT) axons in dorsolateral striatum (DLS) while holding the cell at -70 mV (AMPA) or +40 mV (NMDA). Traces recorded from direct pathway spiny projection neurons (dSPNs) and in indirect pathway SPNs (iSPNs) in zQ175$^{-/-}$ controls (black, zQ175$^{-/-}$) compared to zQ175$^{+/-}$ (blue). Scale bars: 10 ms, 50pA. **(b)** NMDA/AMPA ratios were unchanged in zQ175$^{+/-}$ compared to zQ175$^{-/-}$ in both dSPNs and iSPNs. Mann-Whitney non-parametric tow-sided test (dSPNs zQ175$^{-/-}$ (N=4, n=11), zQ175$^{+/-}$ (N=5, n=16), p = 0.749; iSPNs zQ175$^{-/-}$ (N=4, n=11), zQ175$^{+/-}$ (N=5, n=16), p = 0.058). All mice were male. All Box-plots represent median and interquartile range, whiskers min/max value.

Figure S3 - Pancani et al.

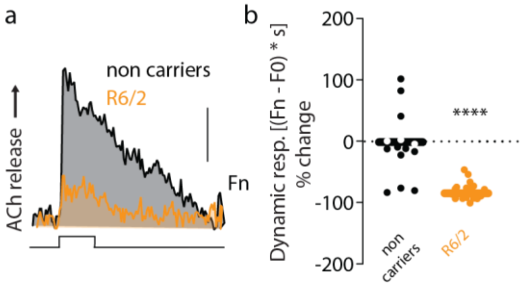

Figure S3 (a) Representative traces showing reduced evoked acetylcholine (ACh) released in R6/2 male mouse model of HD compared to non-carriers controls (scale bar 0.05; Fn: normalized fluorescence). **(b)** Graph shows data summary, % change compared to controls. (non-carriers N=5, n=15 slices; R6/2 N=5, n=18; **** $p < 0.0001$ in a Mann-Whitney non-parametric two-sided test). All mice were male.

Figure S4 - Pancani et al.

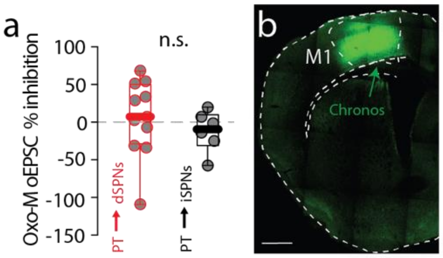

Figure S4 (a) Boxplot summarizing the acute effect of Oxotremorine-M (Oxo-M, 10 μM) on optically evoked EPSCs (oEPSCs) evoked by stimulating pyramidal tract (PT) terminals and monitored during the last 3 min of Oxo-M application in Sim 1 - kj18 PT-cre mice. No different effect was seen in direct and indirect pathway spiny projection neurons (dSPNs vs. iSPNs) in a Mann-Whitney non-parametric two-sided test (p=0.3011; dSPNs (N=5; n=11), iSPNs (N=5, n=6). All Box-plots represent median and interquartile range, whiskers min/max value. **(b)** Confocal image showing a coronal hemi-slice expressing AAV9.hSyn.Chronos-GFP in primary motor cortex (M1) (N=8; Scale bar: 1 mm). All mice were male.

Figure S5 - Pancani et al.

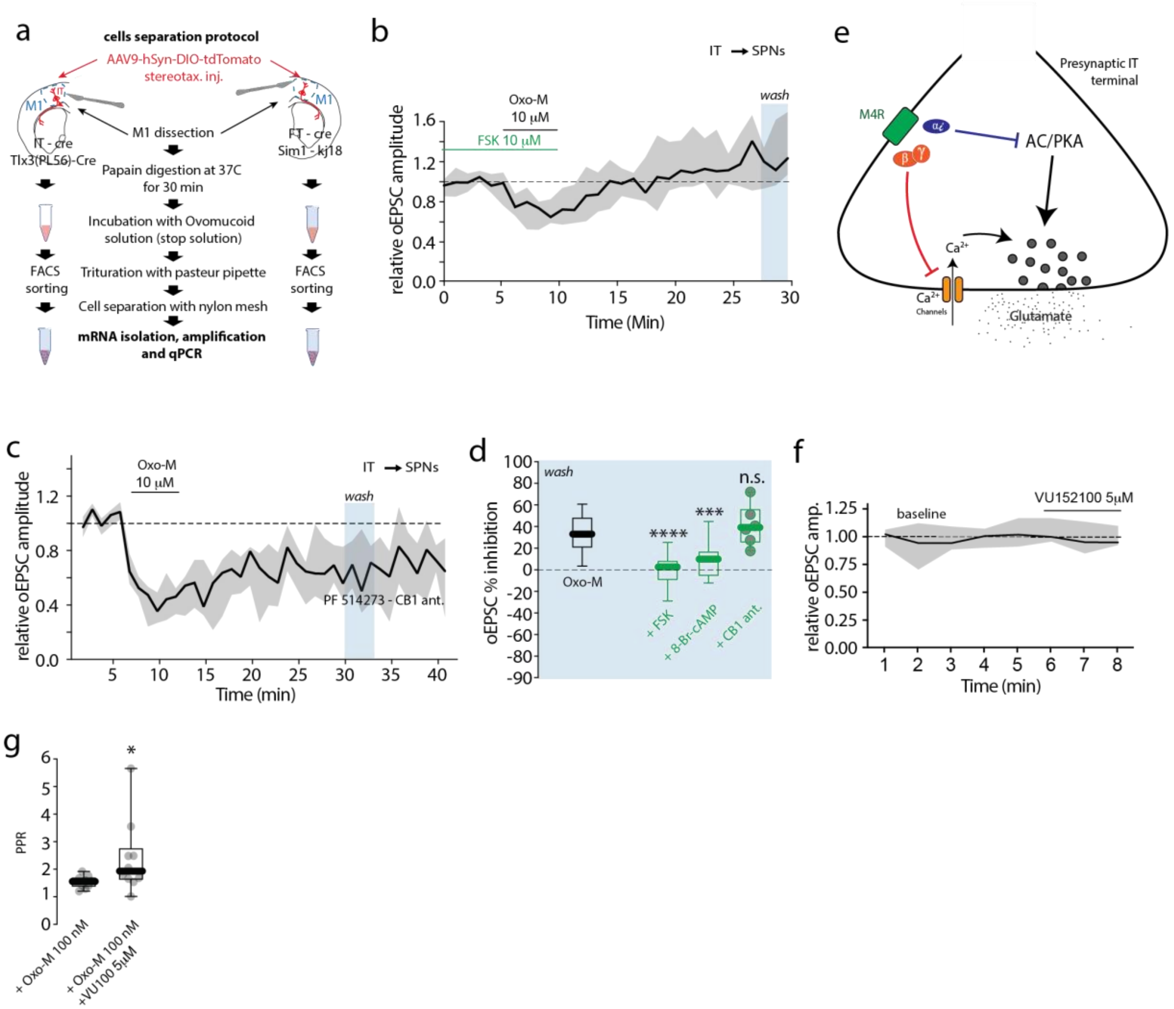

Figure S5 (a) primary motor cortex (M1) tissue expressing td-tomato in IT(PL56-cre) or PT(Sim1-kj18) neurons is removed from freshly cut slices enzymatically separated, FACS-sorted, and analyzed (see Materials and Methods). **(b)** Time-series of optically-evoked EPSCs (oEPSC) amplitudes normalized to baseline control and plotted over time. No Oxotremorine-M (Oxo-M)-induced depression (LTD) at intratelencephalic (IT)-spiny projection neurons (SPNs) synapses when slices were pre-incubated with forskolin (FSK 10 μM) for 20-30 min before Oxo-M application. **(c)** pre-incubation with CB1 antagonist

PF 514273 (1 μM) for 20-30 min. had no effect on the LTD. **(d)** boxplot data summarizing the effect of Oxo-M on oEPSCs during the *wash* period in slices with and without pre-incubation with FSK, 8-Br-cAMP, PF514273. p-values, Kruskal-Wallis with Dunn's multiple comparison test: Oxo-M (N=8, n=22) vs. i) Oxo-M + FSK (N=5, n=12), **** p=0.0001; ii) Oxo-M + 8-Br-cAMP (N=4, n=10), ***p=0.0197; iv) Oxo-M vs. Oxo-M + CB1 ant. (N=2, n=6), n.s. p>0.9999. **(e)** Potential mechanism. Activation of M4 (muscarinic M4) $G_{i/o}$ coupled signaling complement would result in activation of $G_{i\alpha}$ and $G_{i\beta\gamma}$. Our data (Figure **S4b** and **6e**) suggest that the LTD generated by mAChR activation is dependent on M4 inhibition of adenylyl cyclase (AC) and decrease in PKA activity, since it is eliminated by increasing the levels of cyclic AMP (cAMP) by Forskolin or direct activation of PKA with 8-Br-cAMP both applied before and during the induction phase. The long-term effect of Oxo-M would be mediated by $G_{i\alpha}$. Moreover, the transient acute inhibition seen with Oxo-M application is not dependent on PKA activity and could be mediated by $Gi_{\beta\gamma}$ inhibition of voltage gated calcium channels. **(f)** no effect of the M4R positive allosteric modulator (PAM) VU152100 (5μM) on oEPSC amplitude. **(g)** pre-treatment with VU100, increases significantly the paired pulse ratio (PPR) when applied together with a sub-maximal concentration of Oxo-M (100μM) compared to Oxo-M alone, suggesting a pre-synaptic effect of the PAM (*p=0.035 Mann-Whitney non-parametric two-sided test, n=10 neurons for both groups). In b, c, d and f shading represent the interquartile range and line the median. All mice were male. All Box-plots represent median and interquartile range, whiskers min/max value.

Figure S6 - Pancani et al.

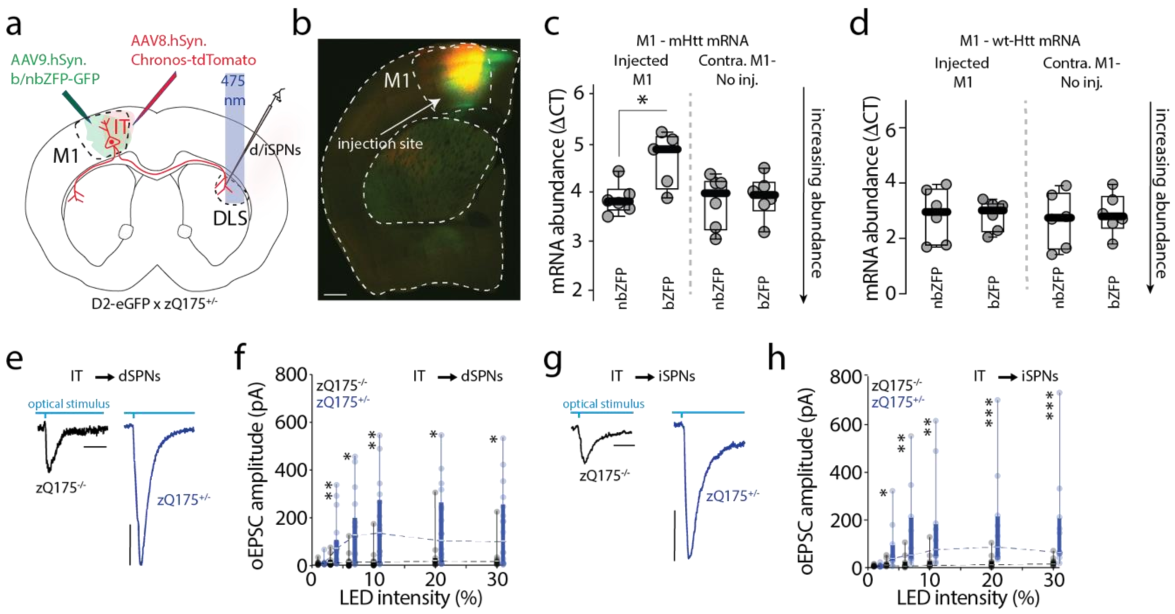

Figure S6 (a) Schematic representation of AAV8.hSyn.Chronos-tdTomato and AAV9.hSyn.bZFP-eGFP constructs being injected in primary motor cortex (M1). Spiny projection neurons (SPNs) recording site in the contralateral dorsolateral striatum (DLS) is also shown. **(b)** Representative confocal image showing co-localization chronos and zinc finger protein (ZFP) constructs in M1 (scales bar 0.5 mm; N=18). **(c)** binding ZFP (bZFP) expression reduced mutant huntingtin (mHtt) mRNA in M1 (evidenced by increased ΔCT values), compared to the nbZFP-injected mice used as controls (*p = 0.0303, Mann-Whitney non-parametric test, M1 tissue from N=5 animals (bZFP), and N=6 (non-binding ZFP (nbZFP)-control), while had no effect on mHtt levels in the contralateral M1 (Contra. M1-No inj., p > 0.05, Mann-Whitney non-parametric two-sided test, M1 tissue from N=5 animals (bZFP) and N=6 (nbZFP). **(d)** bZFP expression had no effect on wildtype-Htt in the injected and contralateral M1 regions (p > 0.05 Mann-Whitney non-parametric two-sided test, M1 tissue from N=6 animals (bZFP), and N=6 (nbZFP-control), and in the contralateral M1 (Contra. M1-No inj., p > 0.05, Mann-Whitney non-parametric test, M1 tissue from N=6 animals (bZFP) and N=6 (nbZFP). **(e-h)** ZFP expression in M1 did not normalize corticostriatal intratelencephalic (IT) transmission in direct dSPNs (**e, f**), and indirect pathway iSPNs (**g, h**). Shown are the representative traces of optically evoked EPSCs (oEPSCs) elicited by stimulating IT terminals and recorded in dSPNs (**e**) and iSPNs (**g**). Graphs summarizing oEPSCs peak amplitude I/O data plotted against LED intensity in dSPNs (median, min/max and interquartile range shown, **f**; wildtype (N=4, n=11) vs. zQ175$^{+/-}$ (N=5, n=18), p-value (LED Intensity, % of max): n.s. (1%),

** 0.0057 (3 %), * 0.0139 (6 %), ** 0.0078 (10 %), * 0.0207 (20 %), * 0.0429 (30 %), and iSPNs (**h**; wildtype (N=4, n=10) vs. $zQ175^{+/-}$ (N=5, n=11), p-value (LED Intensity, % of max): n.s. (1%), * 0.0357 (3 %), ** 0.0021 (6 %), ** 0.0028 (10 %), *** 0.0008 (20 %), *** 0.0006 (30 %)). All mice were male. All Box-plots represent median and interquartile range, whiskers min/max value. Mann-Whitney non-parametric two-sided tests.

Figure S7 - Pancani et al.

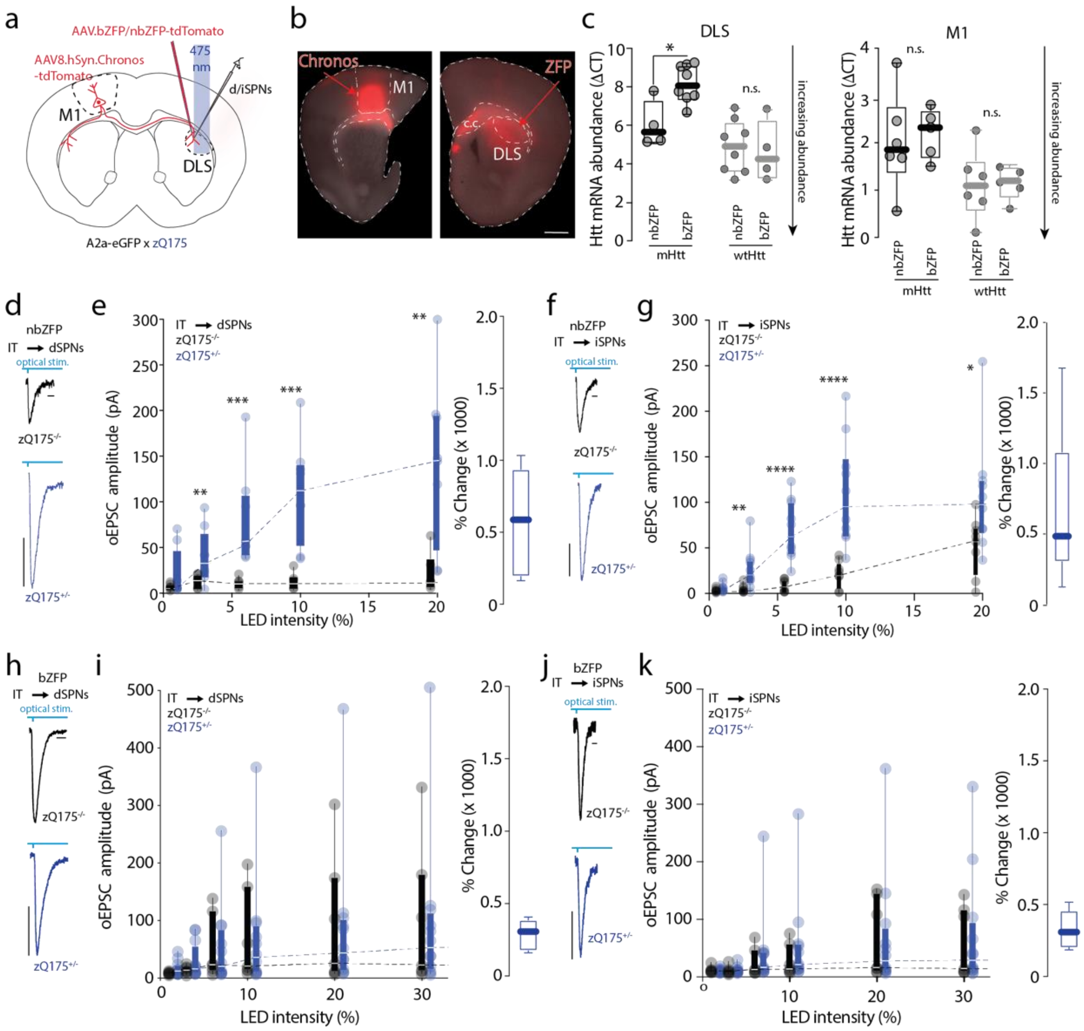

Figure S7 (a) Schematic of AAV8.hSyn.Chronos-TdTomato and AAV.b/nbZFP-tdTomato injected in primary motor cortex (M1) and contralateral dorsolateral striatum (DLS) respectively. **(b)** confocal images of tdTomato fluorescence associated with chronos in M1 (*left*) and ZFP (zinc finger proteins) in contralateral DLS (*right*; Scale bar: 1 mm; N=4). **(c)** binding ZFP (bZFP) expressed in DLS reduced mutant huntingtin (mHtt) mRNA levels compared to non-binding ZFP (nbZFP)-injected mice (*p=0.0485 in a Mann-Whitney no-parametric two-sided test, N = 4 mice (bZFP), N = 8 (nbZFP)), while did not change mHtt mRNA levels in contralateral M1 (n.s., p>0.05 in a Mann-Whitney non-parametric two-sided test). No change in wtHtt neither in DLS nor in M1 (n.s.; grey boxplots; p > 0.05 Mann-Whitney non parametric two-sided test). **(d-g)** nbZFP expression in DLS does not normalize IT-SPNs transmission. Representative traces in **d** (dSPNs) and **f** (iSPNs). Graphs summarizing eEPSCs peak amplitude I/O data plotted against LED intensity in **e** for dSPNs - $zQ175^{-/-}$ (N=3, n = 6) vs. $zQ175^{+/-}$ (N=3, n=8), p-value (LED Intensity, % of max): 0.6377(1%), *0.0400(3%), ***0.0007(6%), ***0.0007(10%), **0.0077(20%), - and in **g** for iSPNs - wildtype (N=3, n=8), $zQ175^{+/-}$ (n=10), p-value (Stim. Intensity, % of max): 0.3254 (1%), **0.0085(3 %), ****<0.0001(6 %), ****<0.0001(10 %), * 0.0343(20 %). All tests are Mann-Whitney non-parametric two-sided test. A group analysis shows no significant difference between dSPNs and iSPNs $zQ175^{-/-}$ data (p=0.693 Mann-Whitney non-parametric two-sided test). **d, g** boxplots on the right showing percentage change of $zQ175^{+/-}$ compared to

controls zQ175$^{-/-}$, at max LED intensity. **(h-k)** bZFP construct in DLS normalized IT-SPNs connectivity. Representative traces are shown in **h** (dSPNs) and **j** (iSPNs). **i**, **k** No difference in input/output responses was seen in cells from zQ175$^{-/-}$ compared to zQ175$^{+/-}$ in both dSPNs (**i**) (zQ175$^{-/-}$ controls (N=3, n=7), zQ175$^{+/-}$ (N=6, n=12), p-value (LED Intensity, % of max): 0.3749 (1%), 0.5380(3 %), >0.9999(6 %), 0.9298(10 %), 0.9298(20 %), > 0.9999(30 %),), and (**k**) iSPNs (zQ175$^{-/-}$ controls (N=3, n=8), zQ175$^{+/-}$ (N=6, n=13), p-value (LED Intensity, % of max): 0.7108(1%), 0.6504(3%), 0.2991(6%), 0.3402(10%), 0.9018 (20%), 0.9017(30%)). All data tested with a Mann-Whitney non-parametric two-sided test. All mice were male. All Box-plots represent median and interquartile range, whiskers min/max value.

Figure S8 - Pancani et al.

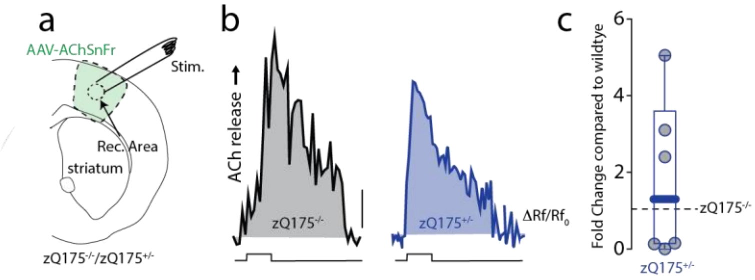

Figure S8 (a) Schematic of AAV.AChSnFr viral construct stereotaxically delivered in primary motor cortex (M1) of zQ175$^{-/-}$ controls or zQ175$^{+/-}$ mice. **(b)** Representative traces showing similar evoked acetylcholine (ACh) released in zQ175$^{+/-}$ compared to zQ175$^{-/-}$ controls. Scale bars: 50 grey values. **(c)** Boxplot summarizing the data showing no change in ACh release in zQ175$^{+/-}$ compared to zQ175$^{-/-}$ controls. Shown is the median from zQ175$^{-/-}$ controls (dashed line) compared to the fold change in zQ175$^{+/-}$. No difference in ACh release is seen in zQ175$^{-/-}$ controls compared to zQ175$^{+/-}$. zQ175$^{-/-}$ controls (N=2 animals, n=7 slices), zQ175$^{+/-}$ (N=2, n=6), p= 0.730 in a Mann-Whitney non-parametric two-sided test. All mice were male. Box-plots represent median and interquartile range, whiskers min/max value.

Figure S9 - Pancani et al.

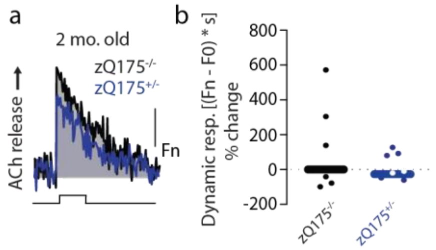

Figure S9 (a) Representative traces showing similar evoked acetylcholine (ACh) released in zQ175$^{+/-}$ compared to zQ175$^{-/-}$ controls in 2 months-old mice (scale bar 0.05). **(b)** No difference in ACh release was seen in zQ175$^{+/-}$ compared to interleaved zQ175$^{-/-}$ controls (data summary % change compared to interleaved littermate zQ175$^{-/-}$ controls, zQ175$^{-/-}$ N=3 mice, n=7 slices; zQ175$^{+/-}$ N=3, n=8; p = 0.5358 in a Mann-Whitney non-parametric two-sided test). Shown is the median (line) and single data points. All mice were male.

Figure S10 - Pancani et al.

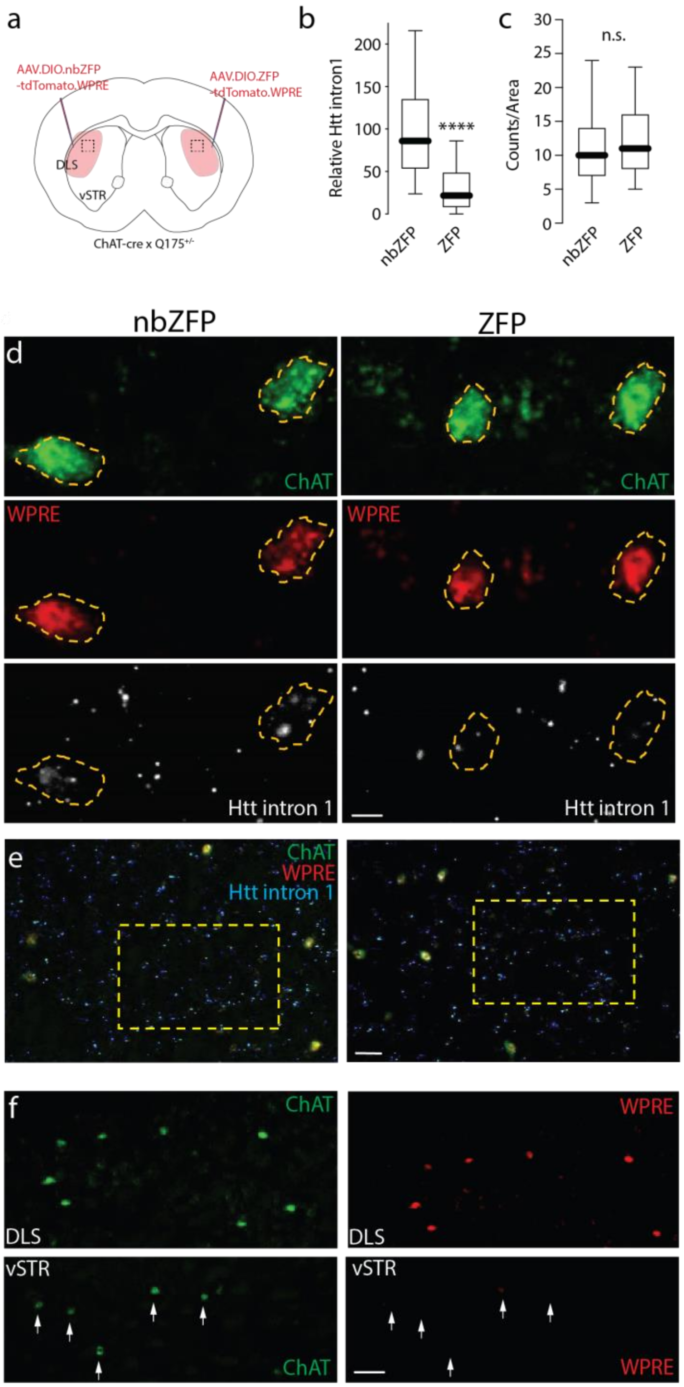

Figure S10 Targeted binding zinc finger protein (bZFP) expression in ChAT+ neurons in dorsolateral striatum (DLS) selectively reduces mutant huntingtin (mHtt) levels. **(a)** Schematics of the ZFP constructs stereotaxic injections and imaging. **(b)** The expression of the AAV.DIO.ZFP-tdTomato-WPRE reduced mHtt levels in ChAT+ neurons in DLS (ZFP panels on the right - as measured by Htt-intron 1 expression; probe details in Material and Methods section) while the non-binding control construct (nbZFP) did not (**b**; **d**, N=5 mice; n=24 DLS areas CTRL-nbZFP, n=22 bZFP; **** p = 0.0001 Mann-Whitney non-parametric two-sided test). Scale bar 10 µm. **(c; e)** we also evaluated the levels of mutant huntingtin in areas of the DLS excluding ChAT+ areas and we saw no difference comparing the ZFP vs. the nbZFP injected areas, suggesting that the mHtt-reducing effects of the bZFP construct is limited to ChAT-cre cells (N=5 mice; n=23 DLS areas CTRL-nbZFP, n=19 bZFP; n.s. p = 0.3296). Scale bar 20 µm. **(f)** ZFP and nbZFP construct was selectively expressed in ChAT+ neurons. Woodchuck Hepatitis Virus Posttranscriptional Regulatory Element (WPRE) included in the AAV constructs is only evident in ChAT-cre neurons in the injection site (**f**, top). Ventra striatum vSTR (with no viral injection, see **a**) does not show WPRE expression (**e**, bottom). Scale bar 50 µm. All mice were male. All Box-plots represent median and interquartile range, whiskers min/max value.

Figure S11 - Pancani et al.

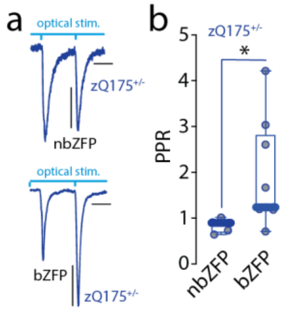

Figure S11 (a) Representative traces of optically evoked EPSCs (oEPSC) recorded in contralateral dorsolateral striatum (DLS) obtained by stimulating intratelencephalic (IT) axons in ChAT-cre x zQ175$^{+/-}$ mice expressing binding/non-binding ZFP (bZFP/nbZFP) selectively in ChAT+ neurons. Figure shows significantly increased paired pulse ratio (PPR) in spiny projection neurons (SPNs) from mice injected with bZFP construct compared to mice injected with the nbZFP control construct - suggesting a presynaptic reduction of excitatory transmission with ZFP expression. **(b)** boxplot summarizing these experiments (n=5 neurons nbZFP; n=9 neuron bZFP; * p = 0.012; Mann-Whitney non-parametric two-sided test; data from Fig. 7 dataset). All mice were male. Box-plots represent median and interquartile range, whiskers min/max value.

Figure S12 - Pancani et al.

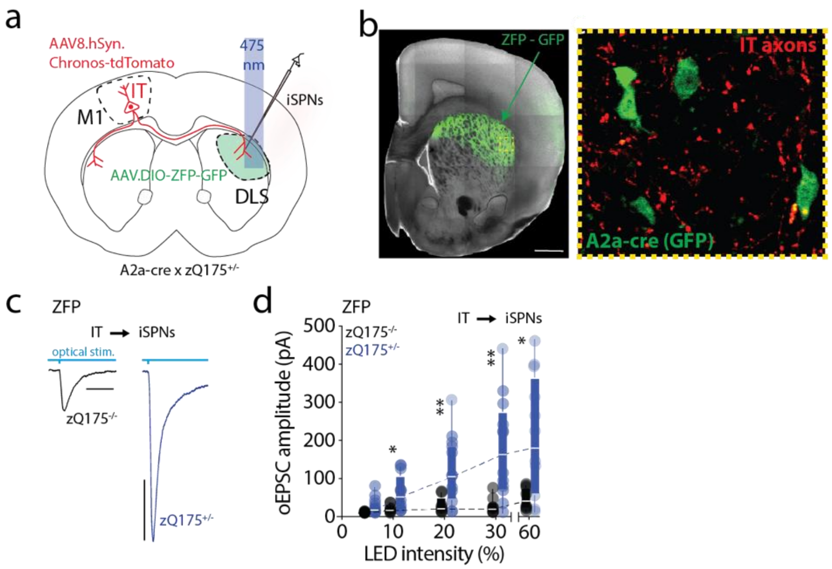

Figure S12 (a) Schematic of AAV8.hSyn.Chronos-TdTomato and AAV.DIO.ZFP-GFP injected in primary motor cortex (M1) and contralateral dorsolateral striatum (DLS) respectively, in A2a-cre x zQ175$^{+/-}$ mice. **(b)** *Left*, confocal image of a coronal slice expressing AAV.DIO.ZFP-GFP in dorsal striatum (Scale bar: 1 mm). *Right*, closeup of the DLS region evidencing A2A-cre neurons (indirect pathway spiny projection neurons, iSPNs) expressing the zinc finger protein associated with GFP (ZFP-GFP) construct together with tdTomato-positive axons. **(c)** Representative traces of optically evoked EPSCs (oEPSCs) recorded in contralateral DLS obtained by stimulating IT axons. Expression of the cre-dependent ZFP construct specifically in iSPNs did not normalize intratelencephalic (IT) corticostriatal transmission onto iSPNs. Data collected only from iSPNs expressing GFP fluorescence (Scale bar: 10 ms and 50 pA). **(d)** Graphs showing increased oEPSCs amplitude when plotted against LED intensity in iSPNs from zQ175$^{+/-}$ mice compared zQ175$^{-/-}$ controls. I/O relationship, zQ175$^{-/-}$ controls (N=3,

n=8 neurons) vs. zQ175$^{+/-}$ (N=3, n=12); p-value (LED Intensity, % of max): *0.0124 (5 %), *0.0124 (10 %), **0.0096 (20 %), **0.0022 (30 %), *0.0473 (60 %), Mann-Whitney non-parametric two-sided tests. All mice were male. All Box-plots represent median and interquartile range, whiskers min/max value.

Figure S13 - Pancani et al.

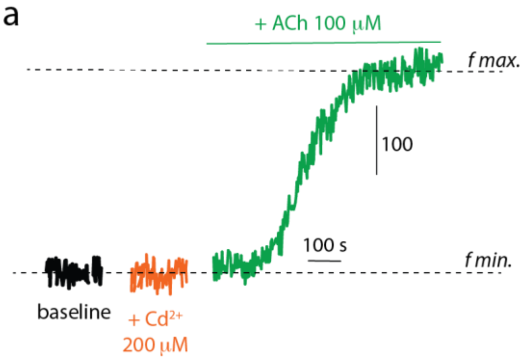

Figure S13 (a) Representative traces of the calibration procedure used for acetylcholine (ACh) release measures (see methods for details). At the end of each experiment AChSnFr (Acetylcholine Sniffer) baseline fluorescence values (*f*) are acquired. 5-minute bath application of Cadmium (Cd^{2+}) 200 μM did not change the fluorescence values recorded (orange), suggesting that there is little (or none) tonic ACh release in the slices (f_{min}). Max fluorescence values of the AChSnFr probe in each slice are subsequently obtained by perfusing ACh 100 μM (green) for a few seconds until a plateau fluorescence is reached (f_{max}). Bar 100 seconds.

Figure S14 - Pancani et al.

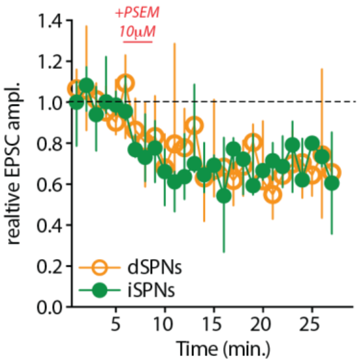

Figure S14 Time series data showing the prolonged effect of pharmacologically selective effector module (PSEM) application (3 minutes bath application) on optically evoked EPSCs (oEPSCs0 in direct pathway spiny projection neurons (dSPNs, tdTomato positive, orange) and putative indirect pathway iSPNs (green). Data from re-plotted Figure 5 (N=4, n=4 dSPNs and n=5 iSPNs). Data shows the median (circle) and interquartile range (bar). All mice were male.

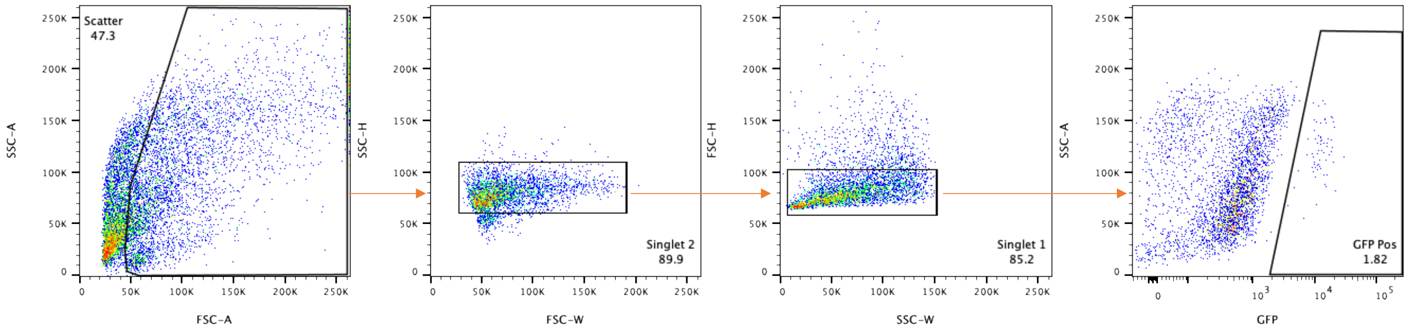

Figure S15 Gating strategy employed for cell sorting. Neurons gated based on intermediate to high forward and side scatter (FSC and SSC) expression and set to exclude debris and non-intact cells. Cell doublets were further excluded using a FSC and SSC -height versus width plots. Enriched single cells were further plotted to identify GFP expression. GFP negative neuron controls were used to set the gates and region, to assess for positive expression.

Led intensity (%of Max)	1	3	6	10	20	30	60
Power (mW)	0.6	1.8	3.6	6	12	18	36

Table S1. Power of illumination associated with the relative LED intensity values used for optogenetic stimulation. Values measured at the objective.
