# Supplementary material for: Cholinergic deficits selectively boost cortical intratelencephalic control of striatum in male Huntington’s disease model mice
Source: Nat Commun. 2023 Mar 14;14:1398. doi: 10.1038/s41467-023-36556-3 (PMC10011605; doi:10.1038/s41467-023-36556-3)
Supplement: Supplementary file 3 — Reporting Summary [file 41467_2023_36556_MOESM3_ESM.pdf]

Corresponding author(s): James Surmeier
Last updated by author(s): Jan 4, 2023

Reporting Summary

Nature Portfolio wishes to improve the reproducibility of the work that we publish. This form provides structure for consistency and transparency in reporting. For further information on Nature Portfolio policies, see our Editorial Policies and the Editorial Policy Checklist.

Statistics

For all statistical analyses, confirm that the following items are present in the figure legend, table legend, main text, or Methods section.

n/a	Confirmed	
☐	☒	The exact sample size (*n*) for each experimental group/condition, given as a discrete number and unit of measurement
☐	☒	A statement on whether measurements were taken from distinct samples or whether the same sample was measured repeatedly
☐	☒	The statistical test(s) used AND whether they are one- or two-sided<br>*Only common tests should be described solely by name; describe more complex techniques in the Methods section.*
☒	☐	A description of all covariates tested
☒	☐	A description of any assumptions or corrections, such as tests of normality and adjustment for multiple comparisons
☐	☒	A full description of the statistical parameters including central tendency (e.g. means) or other basic estimates (e.g. regression coefficient) AND variation (e.g. standard deviation) or associated estimates of uncertainty (e.g. confidence intervals)
☐	☒	For null hypothesis testing, the test statistic (e.g. *F*, *t*, *r*) with confidence intervals, effect sizes, degrees of freedom and *P* value noted<br>*Give P values as exact values whenever suitable.*
☒	☐	For Bayesian analysis, information on the choice of priors and Markov chain Monte Carlo settings
☒	☐	For hierarchical and complex designs, identification of the appropriate level for tests and full reporting of outcomes
☒	☐	Estimates of effect sizes (e.g. Cohen's *d*, Pearson's *r*), indicating how they were calculated

Our web collection on statistics for biologists contains articles on many of the points above.

Software and code

Policy information about availability of computer code

Data collection	pClamp 10 (Molecular Device), Prairie view 5.0 (Brucker inc.), Olympus Fluoview Confocal FW10i, Fiji (mageJ) 2.9.0, FlowJo 10.8.1
Data analysis	Graphpad Prism 9.0, Excel (v16.68), Clampfit 10.0, Fiji (mageJ) 2. 9.0

For manuscripts utilizing custom algorithms or software that are central to the research but not yet described in published literature, software must be made available to editors and reviewers. We strongly encourage code deposition in a community repository (e.g. GitHub). See the Nature Portfolio guidelines for submitting code & software for further information.

Data

Policy information about availability of data

All manuscripts must include a data availability statement. This statement should provide the following information, where applicable:

- Accession codes, unique identifiers, or web links for publicly available datasets
- A description of any restrictions on data availability
- For clinical datasets or third party data, please ensure that the statement adheres to our policy

All data needed to evaluate the conclusions in the paper are present in the paper and/or the Supplementary Materials. All data generated or analyzed in this study are included in this published article (and its Supplementary material files).

Human research participants

Policy information about studies involving human research participants and Sex and Gender in Research.

Reporting on sex and gender	n/a
Population characteristics	n/a
Recruitment	n/a
Ethics oversight	n/a

Note that full information on the approval of the study protocol must also be provided in the manuscript.

Field-specific reporting

Please select the one below that is the best fit for your research. If you are not sure, read the appropriate sections before making your selection.

☒ Life sciences ☐ Behavioural & social sciences ☐ Ecological, evolutionary & environmental sciences

For a reference copy of the document with all sections, see nature.com/documents/nr-reporting-summary-flat.pdf

Life sciences study design

All studies must disclose on these points even when the disclosure is negative.

Sample size	Generally we did not assume a Gaussian distribution for small/moderate sample sizes (~<15) which are prone to type I error with normality tests. Some of the datasets in this manuscript are below a threshold for the computation of a reliable normality test. Therefore we applied non-parametric statistical analyses.
Data exclusions	no data was excluded
Replication	The main findings (the increase in cortico-striatal transmission) were independently confirmed by other researchers and included in the supplementary data. Other on cholinergic signaling in HD was confirmed by an independent study and not included in this paper. Each experiment was performed multiple times across multiple mice/brain slices as explained in the figure legends, and data was pooled for analysis and presentation.
Randomization	Tissue used in the studies were randomly assigned to experimental groups, however, a formal randomization procedure was not implemented.
Blinding	In experiments requiring a comparison between multiple groups (i.e. wt vs. Q175 mice) the investigator was blind to genotype (i.e. wt vs. Q175 mice). Moreover scientific rigor was provided by using multiple models/systems to test our hypothesis (i.e. data repeated in at least three different electrophysiology setup). In time series experiments comparing the effects of a compound (i.e. Oxo-M) before/after drug application on synaptic activity in IT-cre or PT-cre mice the investigator was not blind to mouse genotype as it does not affect result.

Reporting for specific materials, systems and methods

We require information from authors about some types of materials, experimental systems and methods used in many studies. Here, indicate whether each material, system or method listed is relevant to your study. If you are not sure if a list item applies to your research, read the appropriate section before selecting a response.

Materials & experimental systems

n/a	Involved in the study	
☒	☐	Antibodies
☒	☐	Eukaryotic cell lines
☒	☐	Palaeontology and archaeology
☐	☒	Animals and other organisms
☒	☐	Clinical data
☒	☐	Dual use research of concern

Methods

n/a	Involved in the study	
☒	☐	ChIP-seq
☐	☒	Flow cytometry
☒	☐	MRI-based neuroimaging

Animals and other research organisms

Policy information about studies involving animals; ARRIVE guidelines recommended for reporting animal research, and Sex and Gender in Research

Laboratory animals	All animal procedures were performed according to the Northwestern University Animal Studies committee, according to the National Institutes of Health Guide for the Care and Use of Laboratory Animals. Mice were group housed with ad-libitum food and water under a 12 h–12 h light–dark cycle and temperatures of 18–25 °C with 40–60% humidity. Mice used in this study were all male under the C57/B6 background. wild-type and zQ175 +/- heterozygous, ChAT-cre, PT-cre x zQ175, IT-cre x zQ175, ChAT-cre x D1-tdTomato, D1-tdTomato or D2-eGFP x zQ175+/- were all used at 7-9 months of age except where specified otherwise.
Wild animals	no wild animals were used
Reporting on sex	All animals used in this study were male
Field-collected samples	no field-collected animals were used
Ethics oversight	Northwestern University Animal Care and Use Commetee

Note that full information on the approval of the study protocol must also be provided in the manuscript.

Flow Cytometry

Plots

Confirm that:

☒ The axis labels state the marker and fluorochrome used (e.g. CD4-FITC).

☒ The axis scales are clearly visible. Include numbers along axes only for bottom left plot of group (a 'group' is an analysis of identical markers).

☒ All plots are contour plots with outliers or pseudocolor plots.

☒ A numerical value for number of cells or percentage (with statistics) is provided.

Methodology

Sample preparation	Slices from IT or PT neurons expressing tdTomato or eGPF were harvested and processed as described in the supplementary information and Plotkin et al. 2013
Instrument	Neurons were separated on a DB FACSAria SORP system and BD FACSSymphony S6 SORP system
Software	FlowJo 10.8.1
Cell population abundance	Approximately 3000-7000 cells from cortical tissue collected from 2 mice/sample were collected
Gating strategy	Neurons were sorted on a BD FACS Aria SORP instrument equipped with UV, Violet, Blue, Yellow-green and Red laser) using a 100-µm nozzle at 20 psi. Cells were sorted into 300µL solution (Plotckin et al. 2013) for downstream processing. Sample processing and cell sorting was performed in BSL-2 facility using BSL-2 practices. Analysis of the flow cytometry data was performed using FlowJo 10.8.1 software using uniform sequential gating strategy. Neurons were gated based on intermediate to high forward and side scatter (FSC and SSC) expression, and set to exclude debris and non-intact cells. Cell doublets were further excluded using a FSC and SSC -height versus width plots. Enriched single cells were further plotted using a single parameter histogram to identify GFP expression (FITC channel 530/30 band-pass filter, using blue laser excitation). GFP negative neuron controls (processed identical to the specimen), were used to set the gates and region, to assess for positive expression in IT and PT specimen and sort accordingly for qPCR.

☒ Tick this box to confirm that a figure exemplifying the gating strategy is provided in the Supplementary Information.
